# Primary Cutaneous Anaplastic Large Cell Lymphoma With Gastric Metastasis

**DOI:** 10.7759/cureus.32922

**Published:** 2022-12-25

**Authors:** Ayham Khrais, Yazan Abboud, Alexander Le, Param Patel, Sima Vossough-Teehan

**Affiliations:** 1 Department of Medicine, Rutgers University New Jersey Medical School, Newark, USA; 2 Department of Gastroenterology and Hepatology, Rutgers University New Jersey Medical School, Newark, USA; 3 Department of Gastroenterology and Hepatology, East Orange Veterans Affairs Medical Center, East Orange, USA

**Keywords:** extranodal nk/t cell lymphoma, metastatic malignancy, cutaneous t-cell lymphoma, non hodgkin's lymphoma, primary cutaneous lymphomas

## Abstract

Primary cutaneous anaplastic large cell lymphoma (PCALCL) is a subtype of non-Hodgkin lymphoma (NHL) that is localized to the skin. Disseminated disease is rare, and visceral organ involvement is even more so. We report a unique case of PCALCL with gastric metastasis. A 75-year-old man with a history of cutaneous left lower extremity PCALCL status post radiation therapy initially presented with abdominal pain and was found to have diffuse celiac axis and retroperitoneal lymphadenopathy. Endoscopy, initially done to biopsy an involved lymph node (LN), demonstrated a friable gastric nodular lesion with telangiectasias. Biopsy of the lesion and LN revealed anaplastic large cell lymphoma, identical in pathology to the known skin lesion. The patient was treated with systemic chemotherapy with a good response. PCALCL has been thought of as a localized malignancy with a good prognosis and low potential for extracutaneous spread. To our knowledge, this is the first instance of metastatic PCALCL involving the stomach.

## Introduction

Primary cutaneous anaplastic large cell lymphoma (PCALCL) is a non-aggressive monoclonal proliferation of CD30-positive T-cells found within the skin [1,2]. It makes up 25%-30% of cutaneous lymphomas and is the second most common cause of cutaneous T-cell tumors [2]. PCALCL most commonly presents as a slowly growing nodule (or group of nodules) that ulcerates as time progresses. Nodules can regress; however, spontaneous resolution is rare [1]. 

PCALCL has a good prognosis, with a five-year survival rate of 95% [2,3]. This favorable prognosis is thought to be secondary to a multitude of factors, including the expression of skin-specific chemokine receptors on the surface of neoplastic cells, which would limit chemotaxis to the skin itself and reduce the risk of spread [3]. Lower extremity (LE) and extracutaneous involvement, as well as age above 60 years old, are associated with a poorer prognosis. LE involvement specifically is associated with a reduction in the five-year survival rate to 76%, thought to be due to an increased predisposition for regional lymph node spread [3]. 

PCALCL may spread to regional lymph nodes (LNs); however, distant metastasis is rare, with only 10%-13% of cases spreading beyond the integumentary system [1,2]. Gastric spread of cutaneous T-cell lymphomas is especially rare, with only a few documented cases found in the literature [4]. While the stomach is the most common site for primary extra-nodal non-Hodgkin's lymphoma (NHL) (30% of cases), the most common histological subtypes of the disseminated disease usually include diffuse large B-cell lymphoma (45%-59%) and mucosa-associated lymphoid tissue (38%-48%) [5]. Only 1.5%-4% of gastrointestinal NHLs are of the peripheral T-cell lymphoma histological subtype [5]. However, these numbers define primary, not secondary, gastric NHL. The incidence of secondary gastric anaplastic large cell lymphoma is rare, and the spread from PCALCL is virtually unheard of. We present such a case of secondary gastric CD30-positive T-cell lymphoma representing distant metastasis from PCALCL, originating from a lower extremity.

## Case presentation

**Figure 1:**
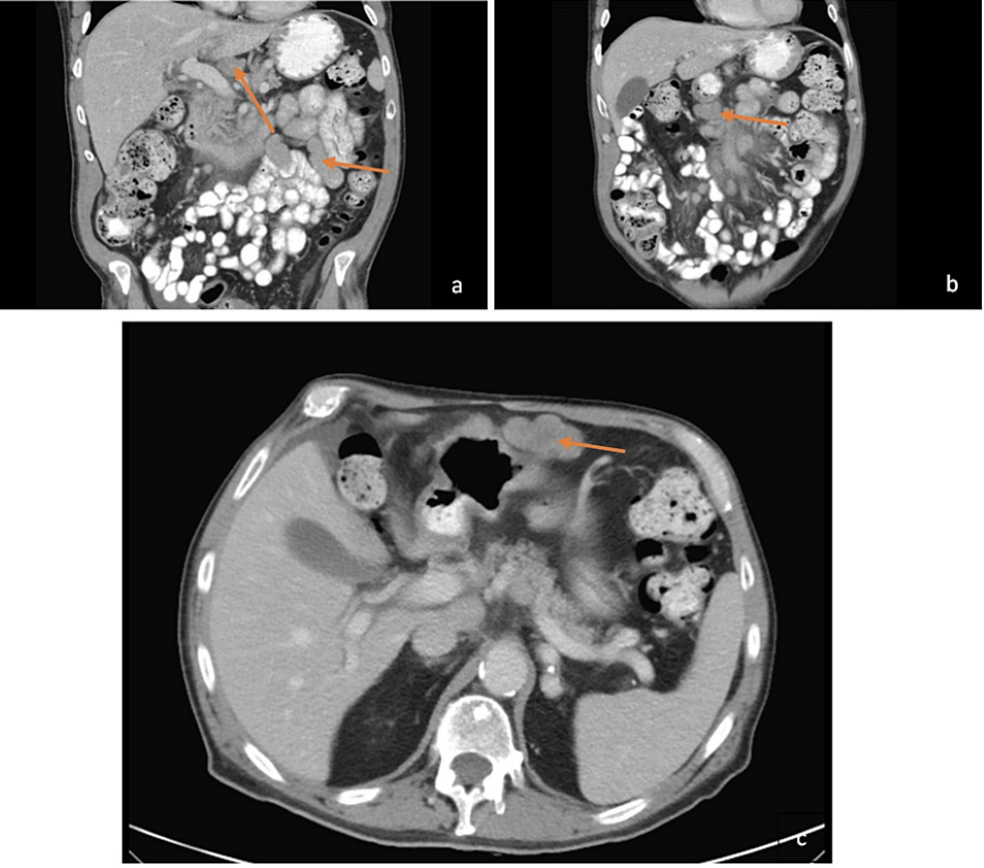
Computed Tomography Scan of the Abdomen and Pelvis With Intravenous Contrast Demonstrating New Celiac Axis and Retroperitoneal Lymphadenopathy (arrows) in the Coronal (a, b) and Transverse (c) Planes.

A 75-year-old man with a history of cutaneous left lower extremity anaplastic T-cell lymphoma status post radiation therapy and coronary artery disease status post placement of three stents presented to the oncology clinic with abdominal pain described as akin to “an iron in [his] stomach.” The patient rated the pain 7-10/10, ameliorated with famotidine but not with the oxycodone he was taking for his lower extremity pain. A computed tomography scan of the abdomen and pelvis with intravenous (IV) contrast was done, revealing new extensive lymphadenopathy in the celiac axis, porta hepatis, and upper retroperitoneal region without bowel obstruction or dilatation (Figure 1). 

**Figure 2:**
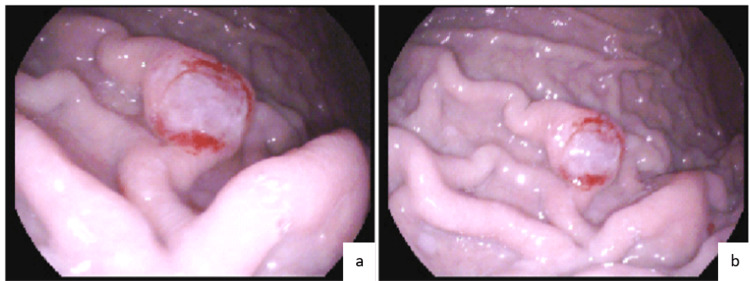
Esophagogastroduodenoscopy Demonstrating a 9-10 mm Friable Gastric Lesion (a, b) With Telangiectasias.

**Figure 3:**
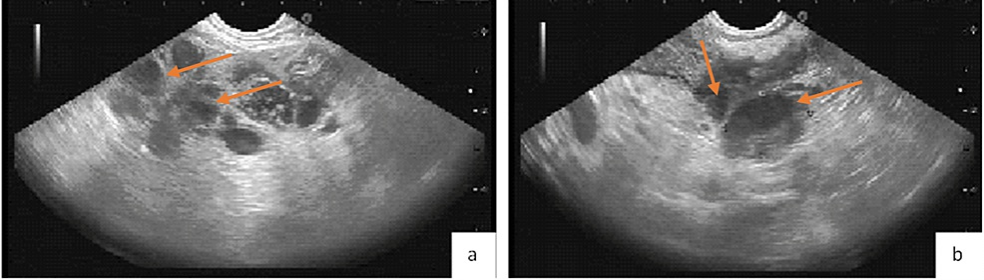
Endoscopic Ultrasound (EUS) Demonstrating a Large Cluster/of Honey Combing Lesions Near the Pancreatic Head (a) and a Matted Cluster of Hypoechoic Lesions Near the Gastric Antrum (b).

**Figure 4:**
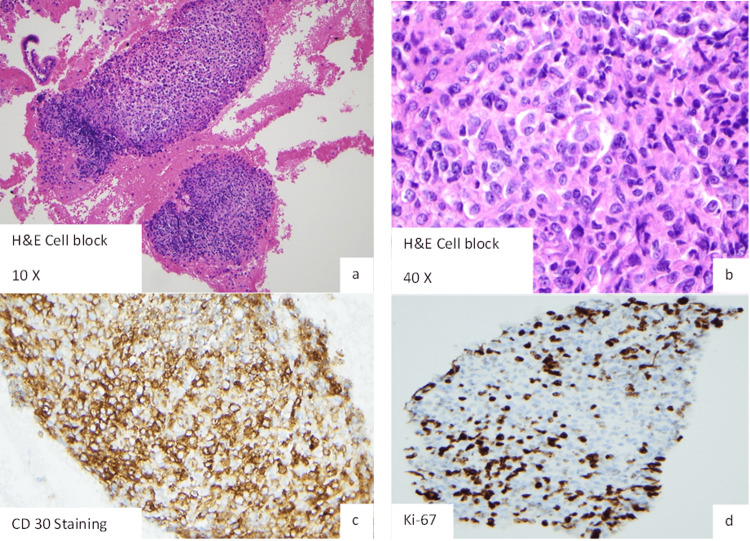
Retroperitoneal Lymph Node Biopsy Demonstrating Clusters of Large Atypical Lymphocyte Proliferation Admixed With GI Contamination (a, b), With Positive CD30 (c) Staining and High (40%) Ki-67 Proliferation (d).

**Figure 5:**
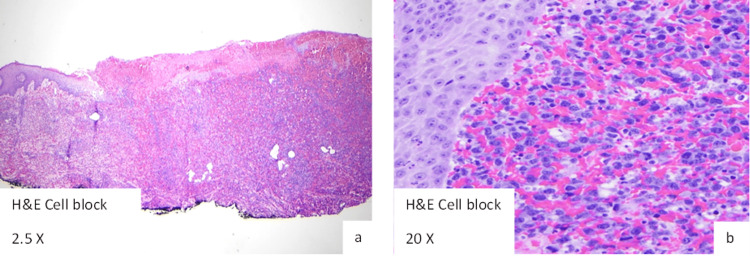
Prior Biopsy of Left Lower Extremity Lesion Demonstrating Anaplastic Large Cell Lymphoma With Positive CD8, CD30, and ALK Staining.

The patient was referred to gastroenterology for esophagogastroduodenoscopy (EGD) with endoscopic ultrasound (EUS) and biopsy of one of the enlarged lymph nodes. EGD demonstrated a 9-10 mm friable gastric nodular lesion with telangiectasias (Figure 2). EUS was significant for a large cluster of hypoechoic lesions near the gastric antrum and pancreatic head (Figure 3). Biopsy of the gastric lesion showed CD30 positive/Bcl-6 positive atypical lymphocytes with enlarged nuclei and prominent nucleoli admixed with GI tract foveolar epithelium (Figure 4), concerning for anaplastic large cell lymphoma. Cells were negative for CD3, CD20, CD45, and pan-cytokeratin. Cells expressed 40% Ki-67 proliferation, and cytoplasm was faintly positive for ALK-1. EUS-guided FNA of the enlarged lymph nodes demonstrated similar pathology as that of the gastric lesion. Both biopsy results were similar in appearance and staining to that of a previous skin biopsy of the known cutaneous anaplastic T-cell lymphoma (Figure 5). The patient was deemed to have gastric metastases of his lymphoma with surrounding lymphadenopathy. He was started on a chemotherapy regimen consisting of brentuximab, cyclophosphamide, doxorubicin, and prednisone (BV-CHP). Restaging CT scan after his fourth cycle of chemotherapy demonstrated near-complete resolution of the previously noted massive abdominal lymphadenopathy.

## Discussion

PCALCL often presents in patients 50 to 70 years of age [1,2]. It initially arises as a solitary cutaneous lesion that varies in appearance from ulcerative to violaceous [2]. Extracutaneous spread is uncommon, with an incidence of 10%, and usually involves regional lymph nodes [2]. Visceral metastases are found in 2% of cases and can occur two months to 10 years after diagnosis [2,6,7]. The low rate of extracutaneous spread is thought to be secondary to the expression of a high concentration of chemokine receptors in malignant cells, including CCR7, CCR8, and CCR10 [8]. Elevated expression of these receptors would maintain localized cellular chemotaxis, preventing distant extension. Before the development of his gastrointestinal symptoms, our patient initially displayed typical symptoms of PCALCL, with one solitary non-ulcerating lower extremity lesion without regional lymphadenopathy or other symptoms. It was initially thought to have responded appropriately to radiation therapy; however, the development of gastric metastasis and retroperitoneal and celiac axis lymphadenopathy indicated distant extracutaneous spread, an unusual clinical manifestation of this disease.

Diagnosis of PCALCL requires biopsy, with pathology revealing large cells ranging in morphology from pleomorphic to anaplastic, with horseshoe-shaped nuclei. Cells often contain eosinophilic, large cytoplasms. Approximately 75% of malignant cells stain positive for CD30 antigen, and negative for CD3 [1,2,6]. ALK staining in PCALCL is rare but portends an increased likelihood of systemic spread [2]. Surrounding the malignant cells are sheets of reactive leukocytes, including lymphocytes, neutrophils and eosinophils. Our patient’s initial skin biopsy of his known left lower extremity lesion demonstrated anaplastic large cell lymphoma staining positive for CD8, CD30 and ALK. T cell receptor beta and gamma PCR results showed monoclonal replication, while fluorescence in situ hybridization (FISH) testing for ALK gene rearrangement was positive. Biopsy of his gastric lesion showed similar atypical lymphocytes with enlarged nuclei and prominent nucleoli; however, these were admixed with GI tract foveolar epithelium, which is dissimilar to skin biopsy findings. Malignant cells from the gastric lesion displayed a similar staining pattern, being positive for CD30 and faintly positive for ALK.

Treatment depends on the extent of the disease and relapse status. Localized lesions are managed with radiation therapy or local excision [2]. Recurrent cutaneous and disseminated lesions are managed with systemic chemotherapy, including methotrexate and brentuximab vedotin (BV) [2]. BV, an anti-CD30 antibody, is the preferred agent for widespread lymph node involvement or visceral disease [2,9]. ECHELON-2, a double-blind, randomized clinical trial, found that the combination of BV with cyclophosphamide, doxorubicin, and prednisone (CHP) was superior to the standard regimen of cyclophosphamide, doxorubicin, vincristine, and prednisone (CHOP) for the management of anaplastic large cell lymphoma with systemic metastasis [9]. Our patient was managed with the combination of BV-CHP and achieved a good systemic response, as indicated in the repeat CT scan showing significant improvement in the widespread lymphadenopathy after four cycles of treatment.

To our knowledge, only one other case of primary cutaneous T-cell lymphoma with gastric metastasis was found in the literature; however, this case depicted mycosis fungoides as the primary lesion [10]. Therefore, this case is unique in that the patient exhibited metastatic spread of a primarily cutaneous disease to a visceral organ, made even more interesting by the fact that there is a lack of literature identifying the involved organ, the stomach, as a target for disseminated PCALCL.

## Conclusions

PCALCL has often been thought of as a limited malignancy confined to the integumentary system and, as such, carries with it a good prognosis. We present a case of a patient with known PCALCL who suffered from abdominal pain, and was subsequently found to have newfound diffuse abdominal lymphadenopathy. Endoscopy, initially performed to biopsy an involved lymph node, discovered a telangiectatic gastric lesion. Biopsy of both the gastric lesion and an involved lymph node demonstrated CD30 (+) PCALCL, similar to a previous biopsy of the patient's known skin lesion. Our patient was treated with a chemotherapy regimen containing Brentuximab with a good response. In patients with a history of PCALCL who present with extracutaneous symptoms, such as abdominal pain, it is important to have metastatic spread with visceral organ involvement on the differential, to promptly rule out such a potentially devastating complication of a usually localized malignancy. Treatment with Brentuximab vedotin, which specifically targets CD30, can result in improvement in such patients. 
